# A study of user switching intention for ERP systems based on push-pull-mooring model: Focusing on the important role of information quality for users

**DOI:** 10.1371/journal.pone.0289483

**Published:** 2023-11-09

**Authors:** Hyeon Jo, Do-Hyung Park

**Affiliations:** 1 HJ Institute of Technology and Management, Bucheon-si, Gyeonggi-do, Republic of Korea; 2 Graduate School of Business IT, Kookmin University, Seoul, Republic of Korea; Sri Sivasubramaniya Nadar College of Engineering, INDIA

## Abstract

Enterprise resource planning (ERP) systems have become indispensable within companies due to their substantial functions and benefits. With a plethora of ERP systems available in the market, management is offered a broad array of options. This paper endeavours to identify the determinants influencing the switching intention of ERP users. Utilizing a conceptual model that adapts the push-pull-mooring paradigm, we seek to construct a formation mechanism of switching intention. Through structural equation modeling conducted on data collected from 236 users, our study uncovers several key findings. The study indicates that system quality, information quality, and top management support exert considerable influence on switching intention via satisfaction. Notably, we found that user satisfaction has a negative impact on switching intention. Our analysis also reveals that alternative attractiveness significantly determines switching intention. These findings provide valuable insights for organizations and ERP vendors to better understand user behaviour and to strategically manage user retention and switching decisions.

## 1. Introduction

Enterprise resource planning (ERP) has become a representative strategic tool for companies. Most medium and large-sized companies use an ERP system because of the advantages such as real-time reports on profitability, sales, and inventory levels [1]. In the early days of ERP distribution, mainly large companies introduced ERP, but now small and medium enterprises (SMEs) are also strengthening their competitiveness with ERP [2]. The global ERP market size is forecasted to reach 86.33 billion $ by 2027, increasing at a CAGR of 9.8% from 2020 to 2027 [3]. As there are many ERP solutions in the market, the choice of executives has expanded. Since recent ERP systems are provided in various forms such as on-premise, cloud, and hybrid, it is simple to install a new system or switch from an existing system to an advanced solution. Despite the growth of the ERP market and the emergence of many alternatives, research on the switching intention between solutions is insufficient.

The importance of understanding the switching intention between ERP systems is becoming increasingly critical for both customers and suppliers in today’s dynamic business environment. For customers, ERP systems represent a significant investment in terms of both time and resources [4]. The decision to switch from one ERP system to another is therefore not one that is taken lightly. It involves considerable costs, including those associated with retraining staff, migrating data, and potential disruption to business operations [5]. Understanding the factors that influence the switching intention can therefore help customers to make more informed decisions about whether to stick with their existing ERP system or to switch to a new one. For suppliers, understanding the switching intention can provide valuable insights into customer dissatisfaction and potential areas for improvement. Customer retention is a key issue for suppliers in the highly competitive ERP market. A high rate of customer defection can not only result in a loss of revenue but can also damage the supplier’s reputation [6]. By understanding the factors that influence the switching intention, suppliers can develop strategies to enhance customer satisfaction and loyalty, thereby reducing the likelihood of customers switching to a different ERP system. In recent years, there has been an increasing interest in the study of the switching intention in the context of ERP systems [7,8]. However, the majority of these studies have focused on the switching intention from an old to a new ERP system within the same supplier [9–11]. The switching intention between different ERP suppliers is an area that has been largely overlooked. This study aims to fill this gap in the literature by investigating the factors that influence the switching intention between different ERP suppliers.

Employees’ perspectives and experiences play a crucial role in ERP adoption and implementation, which justifies our decision to focus on this group. Research suggests that employee satisfaction with an ERP system can significantly impact the overall success of the system within the organization [12]. When employees are satisfied with an ERP system, they are more likely to engage with it effectively, improving the overall performance of the organization [13]. In contrast, when employees have a strong desire to switch to a new and potentially better ERP system, this could indicate dissatisfaction with the current system and could negatively affect their productivity and the organization’s performance. Even though the final decision on switching ERP systems lies with the top management, employees’ switching intention could influence their decision-making process. As such, understanding employees’ intentions to switch ERP systems can provide valuable insights into their level of satisfaction with the current system, potential areas for improvement, and ultimately contribute to the organization’s strategic decision-making regarding its information system infrastructure.

Satisfaction has been validated to significantly affect switching intention in several cases [14–16]. The lower the level of satisfaction of ERP users, the stronger the intention to switch to another system. Thus, this study posits satisfaction as a leading factor affecting switching intention. [17] created an information system (IS) success model to understand the antecedents of net benefits. The model contains three key factors influencing satisfaction. They are system quality, information quality, and service quality. System quality has been found to impact the satisfaction of ERP users [18–20]. Information quality is an imminent driver in enhancing the level of satisfaction in the ERP domains [18,20,21]. Top management support is most vital among critical success factors of ERP [22]. It determines the adoption intention of cloud ERP systems [23]. The more deeply top management engages and supports the ERP implementation process, the better the opinions of users might be reflected. It may also make troubleshooting easier and the degree of user satisfaction would be greater. Therefore, this study postulates that satisfaction is a product of system quality, information quality, and top management support.

Alternative attractiveness serves as the main driver in improving switching intention [24,25]. There is now a wide variety of ERP solutions and managers can select other alternatives easily [3]. The more alternatives there are in the ERP market and the higher the value of the alternatives, the more likely users would intend to switch to other solutions. Hence, this paper suggests that alternative attractiveness is the significant driver of switching intention.

According to migration literature, people would not go to another location because of the influence of some constraints amid strong push and pull effects [26]. Sunk cost moors IS users to current services or solutions [16,27,28]. It refers to the time and effort the user has spent on using the present IS [28]. ERP has many things to learn compared to other simple ISs. Users who have put a lot of effort would hesitate to switch. In this sense, this work verifies the impact of sunk cost on switching intention.

This paper stands out in its innovative approach to understanding the switching intention of ERP users within the context of the push-pull-mooring (PPM) paradigm. While previous research has examined the factors influencing user satisfaction with ERP systems [29–31], less attention has been given to the factors that contribute to the decision of users to switch between different ERP systems. This research gap is critical as understanding the switching intention of ERP users provides invaluable insights for ERP vendors and organizations to enhance user retention strategies, and manage user switching decisions effectively. By combining the PPM paradigm with relevant factors from the information systems success model, this paper presents a comprehensive theoretical model that captures a wider range of potential determinants of switching intention. This is a significant departure from previous studies that have largely focused on a narrow set of determinants. Furthermore, the study utilizes a large sample of users from a significant shipbuilding and marine company, offering a unique context that has not been extensively explored in previous research. The findings from this context can yield valuable implications for similar manufacturing industries. In conclusion, this paper makes a unique contribution by introducing a comprehensive model to understand switching intentions in ERP users, focusing on a unique industry context, and addressing a significant research gap in the literature. Through this, the paper paves the way for more nuanced and context-specific understandings of user behavior in relation to ERP systems.

The Literature Review section describes the related works. The Research Framework Section offers the conceptual framework and each hypothesis. The Empirical Methodology Section covers the empirical methodology and sample information. The Analysis and Results Section contains the results of the structural equation modeling. The Conclusion Section describes the implications, limitations, and future research directions.

## 2. Literature review

### 2.1. ERP

ERP system has been continuously developed and upgraded since its first appearance [32,33]. At the time of the initial formation of the ERP market, it was a tool to secure a competitive advantage [34,35]. However, it is now recognized as a basic IS that is essential even for small and medium-sized enterprises [2]. Accordingly, several ERP models have been released in the corporate IS market, and the range of choices for companies has expanded [3]. Following the ERP trend, many studies have investigated the success factors and mechanisms for transition or adoption to new types such as cloud-based [8,9,18]. Chaveesuk and Hongsuwan [18] found that system quality and information are the predominant determinants of satisfaction of ERP users. Wibowo and Sari [20] confirmed that when the system quality and information quality increase, user satisfaction also improves. Liang, Saraf [36] empirically demonstrated the importance of top management involvement in promoting the degree of ERP use. After the cloud model was developed and released, studies targeting cloud ERP have been conducted. AlBar and Hoque [23] state that top management support is the imperative antecedent of ERP adoption. Chang and Hsu [8] approached the switching process to cloud ERP in terms of benefits and costs. Privacy concerns undermined the transition intentions.

Although research on ERP has been actively conducted for the past several years, there is a scant study on switching intentions in the current mature market. In the ERP market, 1) there are some suppliers, 2) various models have been released, and 3) it is provided in the cloud manner, making it easier for companies to move between systems as it is easier to switch to and install a new system. Therefore, the switching intention between ERP becomes a very important issue for customers or suppliers. This study identifies the satisfaction with the current ERP system based on the previously verified constructs and then explains the intention to switch.

### 2.2. PPM model

The PPM model, originally conceptualized in the field of migration studies, has emerged as a highly applicable framework in the domains of ISs and marketing [24]. The PPM model elucidates the factors influencing individuals’ decisions to migrate from one context to another, be it a geographic location or a service platform [37].

The model articulates that the process of migration is influenced by three types of factors: push, pull, and mooring. Push factors encompass the elements that motivate individuals to leave their existing situation [28,38]. In the realm of IS and marketing, these factors have been identified as dissatisfaction, lack of trust, and insufficient community support [14,28].

Pull factors, on the other hand, are those attributes that attract individuals towards a new environment or service [39]. These factors can range from superior living conditions in the context of geographical migration to improved service quality, attractive alternatives, social influence, and the presence of a critical mass in the realm of service switching [40,41].

Mooring factors, the third element in the PPM model, refer to the individual or societal factors that may either hinder or facilitate the decision to migrate [24,39]. These factors include personal attitudes, subjective norms, and both setup and continuity costs [24,42].

The PPM model has been extensively applied in IS and marketing literature to elucidate the drivers of customer switching behavior, particularly in the context of service migration [28,43,44]. However, as this field continues to evolve, it is crucial to continually revisit and update our understanding of the PPM model and its application. This study aims to contribute to this ongoing dialogue by examining the push, pull, and mooring factors influencing the switching intention of ERP users.

PPM model is considered a preeminent paradigm in the field of migration [24]. The PPM mechanism proposes that the push, pull, and mooring factors have an impact on migrants’ decision to relocate from one geographic location to another. People are pushed away from their initial realm by push factors. [28,38]. Loss of work, ethnic loyalties, and a lack of possibilities for personal growth are examples of push factors [40]. In IS and marketing field, numerous studies have employed satisfaction, trust, and community support as push factors [14,28]. Pull factors entice people to travel to new places [39]. Pull factors could include improved living conditions, higher income, and career prospects. [40]. Several works on switching intention have validated the effects of alternative attractiveness, peer influence, and critical mass as pull factors [41]. Mooring factors are concerned with personal and societal difficulties [24]. These variables can impede or facilitate migration decisions [39]. Mooring factors could contain attitude, subjective norm, setup cost, and continuity cost [24,42].

PPM model has been used in IS and marketing literature to explicate what can cause customers to transfer from a current service to another one [25,28,43].

### 2.3. Switching intention

Switching intention is one of the major topics in consumer behavior or IS contexts such as online games and SNSs [45,46]. A vast body of research has verified that satisfaction enhances the level of switching intention in several domains [14,28]. Several researchers have revealed that alternative attractiveness enhances the level of switching intention [16,25,41]. Empirical evidence on switching behaviors shows that sunk cost is an indispensable antecedent of the switching intention of users [16]. [47] examined the decision factors affecting consumer switching intention in the domain of mobile devices. They figured out that easiness, product usefulness, and relative advantages are the dominant drivers of switching intention. It was also discovered that the biggest obstacle preventing consumers from converting to new technologies was the financial cost. According to Quoquab, Mohammad [15], switching intention is influenced by satisfaction, costs, and customer innovativeness in the telecommunication industry. Asimakopoulos and Asimakopoulos [48] investigated the impact of usability and switching costs on switch intention in the domain of forecasting support systems. They uncovered that switching intention is negatively influenced by both perceived usability and switching costs. Salo and Makkonen [49] postulated that trialing behavior predicts switching behavior among mobile app users. They argued that dissatisfaction, limited recovery resources, and the unfinished nature of apps enable switching intentions. Liang, Choi [14] explored the association between satisfaction, switching intention, and repurchase intention in sharing economy. They unveiled that transaction-based satisfaction, experience-based satisfaction, and trust in hosts harm the formation of switching intention. Chang and Hsu [8] investigated the influencing factors of switching intention to cloud ERP. They demonstrated that easiness, usefulness, and privacy concerns influence switching intention. Although the ERP system has become an essential IS for company activities and there are many alternatives, studies on switching intention are insufficient.

## 3. Research framework

**Fig 1:**
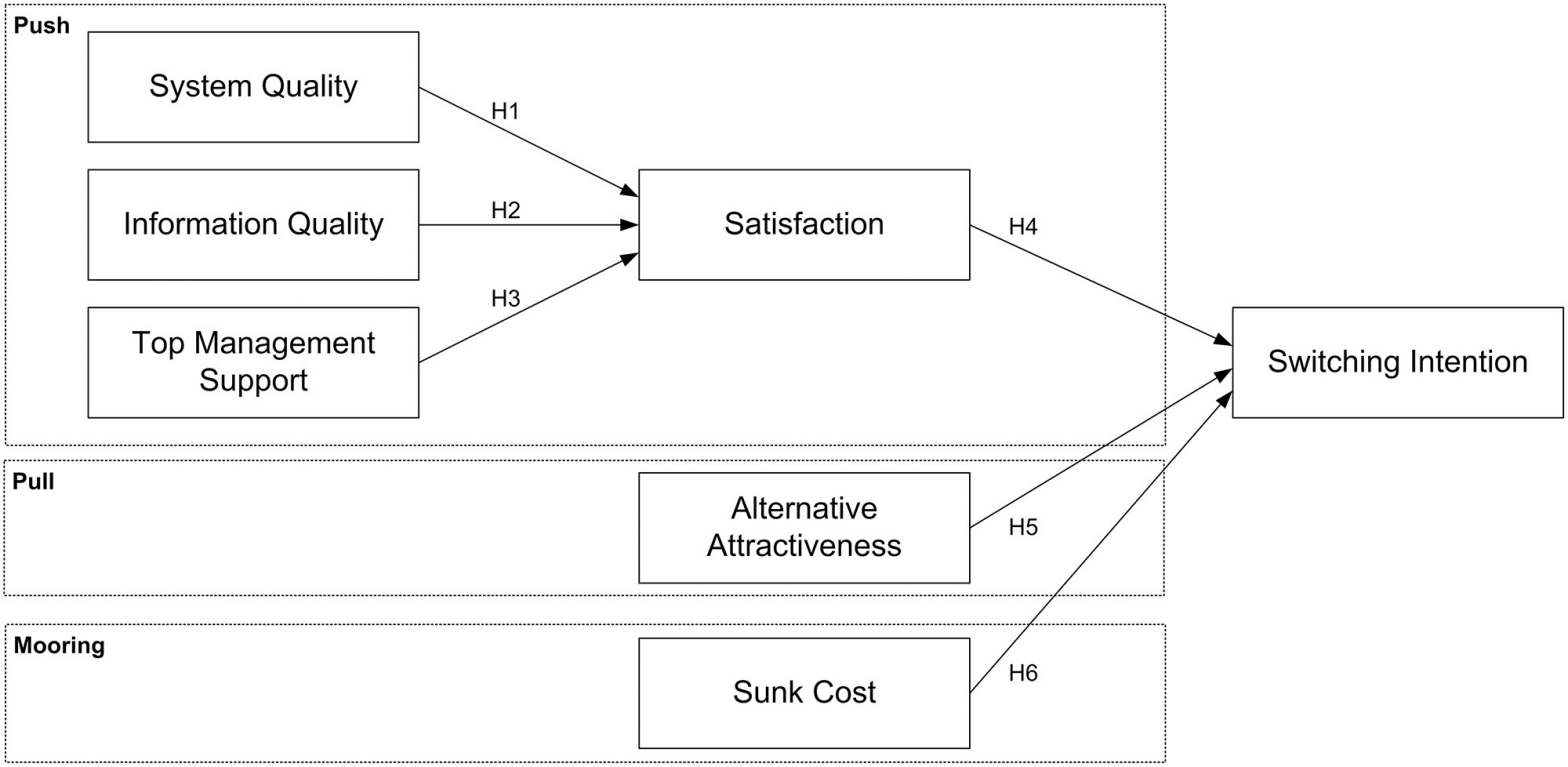
Research model.

Fig 1 illustrates the analytical model for exploring the effects of the enablers and inhibitors on the formation processes of switching intention of ERP users. This paper considers satisfaction, system quality, information quality, and top management support as pushing factors; alternative attractiveness as a pulling variable; and sunk cost as a mooring component.

### 3.1. System quality

System quality, which refers to the desired characteristics of an information system, including reliability, flexibility, ease of use, and response time [17], has been widely acknowledged as a crucial factor influencing user satisfaction in ERP systems [31,50–53]. When an ERP system exhibits high system quality, offering efficient, reliable, and user-friendly functionalities that align with user needs, it tends to enhance user satisfaction [54–56]. Users perceive satisfaction when the ERP system effectively supports their work processes and enables them to achieve their goals [57,58]. Therefore, enhancing system quality is expected to lead to increased user satisfaction with the ERP system [59,60]. Conversely, if the ERP system is unstable and slow, users may experience dissatisfaction and consider switching to an alternative ERP system. Based on these considerations, this study proposes the following hypothesis:

Hypothesis 1. System quality positively impacts satisfaction.

### 3.2. Information quality

Information quality refers to the accuracy, timeliness, relevance, and understandability of information provided to users [61]. Previous studies have consistently highlighted the positive relationship between information quality and user satisfaction [62,63]. For example [64], included information quality as a key factor in their updated IS success model, emphasizing its significant influence on user satisfaction. Furthermore [65], found that users who perceive the information provided by an ERP system to be of high quality tend to exhibit higher levels of satisfaction with the system. Moreover, several authors have corroborated that information quality positively influences the perception and behaviors of IS users in various contexts, such as factory systems [66], social networking [67], e-commerce [68], and e-learning [69]. In particular, [50] confirmed the critical role of information quality in determining user satisfaction in the context of ERP systems. Additionally, studies have shown that information quality positively influences perceived usefulness, which subsequently impacts users’ intention to switch ERP systems [7]. ERP systems provide users with various information about the organization, and the relevance and reliability of this information contribute to user satisfaction. Based on these findings, it is reasonable to hypothesize that the information quality of an ERP system has a positive impact on user satisfaction. Therefore, this study proposes the following hypothesis:

Hypothesis 2. Information quality positively impacts satisfaction.

### 3.3. Top Management support

Top management support refers to the active involvement of senior executives in project implementation, including resource allocation, policy setting, and overall endorsement [70]. Several studies have highlighted the crucial role of top management support in user satisfaction [63,71–73]. For example, Al-Mashari, Al-Mudimigh [74] found that top management support in ERP implementation significantly enhances user satisfaction. Similarly, Umble, Haft [75] demonstrated that strong top management support leads to successful ERP implementations and higher user satisfaction. Recent research by Bradford and Florin [76] also supported this relationship, indicating that top management support creates a supportive environment for ERP use, influencing user satisfaction. As top management support for ERP implementation increases, ERP users are likely to experience higher satisfaction with the system. Based on these findings, it is reasonable to propose that top management support positively influences user satisfaction with ERP systems. Therefore, this study suggests the following hypothesis:

Hypothesis 3. Top management support positively impacts satisfaction.

### 3.4. Satisfaction

Satisfaction is a key determinant of user behavior towards information systems, including ERP systems. It is defined as a user’s overall evaluation of the performance of an information system [64]. It also represents the degree to which users feel that the accessible IS has met their needs for transformation [77]. The relationship between satisfaction and switching intention has been extensively examined in the literature, with general consensus indicating a negative relationship between the two [5,42,78,79]. This suggests that users who are more satisfied with their current ERP system are less likely to consider switching to a different system. Bhattacherjee’s expectation-confirmation model (ECM) also supports this perspective, suggesting that if users’ experiences with a system meet or exceed their expectations, resulting in high levels of satisfaction, they are likely to continue using the system [5]. Similar conclusions were drawn by [78] who found that satisfaction significantly reduced the intention to switch from an existing ERP system. If employees are dissatisfied with the current ERP, they may try to decrease their use or find alternatives. Since ERP systems are essential tools for modern enterprises, they might be willing to switch to other alternatives. Thus, this study suggests the following hypothesis:

Hypothesis 4. Satisfaction negatively impacts switching intention.

### 3.5. Alternative attractiveness

Alternative attractiveness, defined as the perceived value of other available options relative to the currently used system, has been identified as a significant factor in user behavior towards information systems [80]. [81] suggest that alternative attractiveness plays a critical role in shaping users’ attitudes towards their current system, often acting as a pull factor leading to system switching. Furthermore, several studies in the context of IT platforms have found that the attractiveness of alternatives significantly influences users’ switching intentions [25,82]. With the rapid growth of ERP systems, users are presented with numerous alternatives in the IS market. If there are more attractive options, users would tend to consider switching. Moreover, the greater the perceived benefits of an alternative system, the higher the likelihood of users considering a switch. Consequently, when users perceive other systems as offering superior value or utility compared to their current ERP system, they are more likely to consider switching. Thus, this study proposes the following hypothesis:

Hypothesis 5. Alternative attractiveness positively impacts switching intention.

### 3.6. Sunk cost

Sunk cost, which refers to investments that have already been made and cannot be recovered, has been recognized as a significant factor in decision-making [83]. In the realm of information systems, sunk costs encompass the time, effort, and financial resources expended on system adoption and use [16,81,84]. Previous studies have indicated that sunk costs create a psychological barrier, known as the "sunk cost effect," that hinders the abandonment of ongoing projects or the switch to alternative systems [85,86]. Users are less likely to switch to alternative systems, even when those alternatives offer greater value or benefits, due to the perceived waste of prior investments [87]. In the context of ERP systems, employees invest considerable effort into installing the software and learning its functions, leading to a reluctance to switch. This study proposes the following hypothesis in light of these observations:

Hypothesis 7. Sunk cost negatively impacts switching intention.

## 4. Empirical methodology

### 4.1. Measurements

The current work surveyed ERP users to verify the research model. To confirm the validity of the indicators of each factor, the measures were drawn from extant studies in ISs and migration theory. This research slightly modified the measurement indicators to fit the context of ERP. A 7-point Likert scale with the words "strongly disagree" (1) and "strongly agree" (7) was used to evaluate the 24 items. Academic and professional experts in the IS and migration fields thoroughly revised the questionnaire before it was finalized, ensuring that the material was accurate and that the questions were organized logically. In Appendix A, a list of the measurement items and references is provided.

### 4.2. Sample

Employees that used ERP systems in their workplace were the target of this research. While the decision to switch ERP systems is indeed a significant managerial one, it would be also influenced by users’ perceptions and experiences, including those of clerks, assistant managers, and managers. These individuals, as frequent users of ERP systems, are essential informants of system quality, user satisfaction, and switching intentions. Their opinions and experiences can shape the broader organizational perspective on whether to continue or discontinue using an ERP system [88]. In this study, the respondents used a comprehensive and integrated ERP system specifically designed for large-scale shipbuilding and marine engineering companies. The ERP system, provided by a leading global ERP vendor, includes modules that cover various aspects of the company’s operations, including procurement, project management, production planning, quality management, financial accounting, and human resources management. This ERP system is highly customizable, allowing it to be tailored to the specific needs and processes of the shipbuilding and marine engineering industry. It supports both project-based and process-based manufacturing modes, which are commonly used in this industry. Additionally, it includes features that address the unique challenges of the industry, such as complex supply chains, stringent quality requirements, and large-scale project management. The system is deployed on-premise and is accessed by the employees through a secure intranet connection. It is integrated with other software applications used by the company, such as computer-aided design (CAD) and project management tools, to provide a seamless and efficient work environment. The respondents, as employees of the company, use the ERP system on a daily basis for a variety of tasks, such as updating project status, checking inventory levels, issuing purchase orders, and generating financial reports. Their roles and responsibilities within the company determine the specific modules and features of the ERP system they use. Overall, the ERP system used by the respondents is representative of the advanced, industry-specific ERP systems commonly used in large shipbuilding and marine engineering companies.

The respondents received an email with a link to the survey online. Additionally, paper-and-pencil survey forms were distributed. A preliminary data analysis was done after the data collection to filter untruthful responses. A total of 236 replies were used for the analysis. In this work, the minimum need for multiple regression was checked using an a-priori sample size calculator [89]. The minimum necessary sample size is 112 when all the necessary data are entered, including the anticipated effect size of 0.1, the desired statistical power level of 80%, and the probability level of 0.05. This criterion is satisfied since the sample size of this work is 236.

Table 1 presents the demographic information of participants. 209 (88.6%) informants were male and 27 (11%) informants were female. The majority of users were in their 30s and 41.9% of participants were 40s. 68 respondents (28.8%) have worked for over 21 years and 171 respondents (72.5%) have used ERP systems for over 5 years. The positions include clerk, assistant manager, manager, etc. The most used modules in ERP systems were various from operation management to financial accounting.

**Table 1:** Demographics information of respondents.

Demographics	Item	Subjects (N = 236)	
		Frequency	Percentage
Gender	Male	209	88.6
	Female	27	11.4
	Total	236	100.0
Age	10s	1	0.4
	20s	5	2.1
	30s	96	40.7
	40s	99	41.9
	50s	35	14.8
	Total	236	100.0
Working Period	5 years or shorter	24	10.2
	6 years–10 years	53	22.5
	10 years–15 years	38	16.1
	15 years–20 years	53	22.5
	21 years or longer	68	28.8
	Total	236	100.0
Usage Period	5 months or shorter	6	2.5
	6 months–1 year	6	2.5
	1 year–2 years	10	4.2
	2 years–5 years	43	18.2
	6 years or longer	171	72.5
	Total	236	100.0
Position	Clerk	20	8.5
	Assistant Manager	56	23.7
	Manager	41	17.4
	Deputy Manager	67	28.4
	General Manager	45	19.1
	Executive Director	7	3.0
	Total	236	100.0
Modules	Operation Management	28	11.9
	Establish	29	12.3
	Sales/Business Management	11	4.7
	Materials/Procurement	37	15.7
	Quality Management	32	13.6
	Management/Human Resource	32	13.6
	Finance Accounting	39	16.5
	Others	28	11.9
	Total	236	100.0

## 5. Analysis and results

This study analyzed the data through the partial least squares (PLS) method based on structural equation modeling (SEM). PLS provides the advantage of having fewer restrictions on the distribution of sample size and residuals than SEM of covariance techniques such as LISREL and AMOS [90]. To analyze the data, this study used SmartPLS program [91,92]. A two-stage approach was performed to evaluate the measurement model and structural model.

### 5.1. Common Method Bias (CMB)

Given the nature of our study, we recognized the potential risk of CMB as we used a single source (survey responses) to gather our data. CMB could potentially inflate or deflate the reported relationships between constructs if not adequately addressed. We applied Harman’s single-factor test to assess the extent of CMB [93]. The result showed that the most substantial variance explained by a single factor was 42.189%, which is below the critical threshold of 50%, suggesting that CMB is not a significant issue in our study. Additionally, we examined the multicollinearity between constructs by analyzing the variance inflation factor (VIF) values in the inner model. The VIF values for all the constructs (SYQ → SAT: 1.772, INQ → SAT: 2.036, TMS → SAT: 1.698, SAT → SWI: 1.170, ALA → SWI: 1.036, SUC → SWI: 1.160) are less than the stringent threshold of 3, further suggesting that multicollinearity is not a significant issue [94]. These measures, combined with the below-threshold single factor variance, provide reasonable confidence that our results are not significantly distorted by CMB.

### 5.2. Measurement model

To assess the measurement model, reliability and validity were analyzed. The reliability of data was evaluated based on the Cronbach’s alpha coefficient and composite reliability (CR). Cronbach’s alpha and CR estimates of all of the constructs exceeded the recommended threshold value of 0.7 (Nunnally), ensuring the presence of reliability. Validity was checked by considering convergent and discriminant validity. Convergent validity was confirmed by examining both the average variance extracted (AVE) and the factor loadings of the indicators. AVE values ranged from 0.715 to 0.928, which are well over than the accepted limit of 0.5 [95]. The factor loadings ranged from 0.756 to 0.970 and are all statistically significant at the p = 0.001 levels, indicating the satisfactory level of convergent validity [96]. Table 2 describes the construct, items, mean, standard deviation, factor loading, Cronbach’s alpha, CR, and AVE.

**Table 2:** Scale reliabilities.

Construct	Items	Mean	St. Dev.	Factor Loading	Cronbach’s Alpha	CR	AVE
System Quality	SYQ1	4.682	1.304	0.896	0.809	0.889	0.723
	SYQ2	4.280	1.365	0.878			
	SYQ3	4.419	1.285	0.756			
Information Quality	INQ1	4.860	1.289	0.861	0.812	0.889	0.728
	INQ2	4.674	1.282	0.910			
	INQ3	4.068	1.354	0.781			
Top Management Support	TMS1	5.017	1.334	0.930	0.933	0.957	0.882
	TMS2	4.860	1.335	0.933			
	TMS3	4.975	1.308	0.947			
Satisfaction	SAT1	4.297	1.383	0.970	0.963	0.957	0.931
	SAT2	4.225	1.395	0.965			
	SAT3	4.250	1.363	0.956			
Alternative Attractiveness	ALA1	4.504	1.091	0.807	0.880	0.927	0.809
	ALA2	4.593	1.205	0.947			
	ALA3	4.669	1.186	0.933			
Sunk Cost	SUC1	4.436	1.214	0.944	0.940	0.960	0.890
	SUC2	4.377	1.241	0.950			
	SUC3	4.360	1.212	0.937			
Switching Intention	SWI1	4.212	1.234	0.937	0.927	0.954	0.873
	SWI2	4.093	1.265	0.929			
	SWI3	4.203	1.292	0.935			

In this study, discriminant validity was evaluated using both the Fornell-Larcker Criterion and the Heterotrait-Monotrait ratio of correlations (HTMT). The Fornell-Larcker criterion asserts that the square root of the average variance extracted (AVE) for each construct should be greater than its highest correlation with any other construct. As shown in Table 3, the diagonal values (square root of AVE for each construct) are greater than the inter-construct correlations in their respective rows and columns, confirming discriminant validity [95]. Additionally, we used the HTMT method, which requires an HTMT value less than 0.85 for discriminant validity to be established. As shown in Table 4, all HTMT values are well below the 0.85 threshold, except for the relationship between alternative attractiveness and switching intention which is 0.735, still within acceptable range [97].

**Table 3:** Fornell-larcker criterion.

Construct	1	2	3	4	5	6	7
1. System Quality	0.850						
2. Information Quality	0.635	0.853					
3. Top Management Support	0.533	0.614	0.939				
4. Satisfaction	0.667	0.710	0.557	0.965			
5. Alternative Attractiveness	0.158	0.171	0.216	0.167	0.900		
6. Sunk Cost	0.366	0.345	0.315	0.363	0.138	0.943	
7. Switching Intention	0.009	-0.052	0.076	-0.051	0.674	-0.038	0.934

**Table 4:** HTMT.

Construct	1	2	3	4	5	6	7
1. System Quality							
2. Information Quality	0.776						
3. Top Management Support	0.601	0.698					
4. Satisfaction	0.740	0.796	0.579				
5. Alternative Attractiveness	0.213	0.222	0.242	0.202			
6. Sunk Cost	0.408	0.399	0.333	0.387	0.166		
7. Switching Intention	0.065	0.064	0.085	0.055	0.735	0.039	

Taken together, these results provide strong evidence of discriminant validity for our constructs, indicating that they are distinctly different and measure unique phenomena in the context of our research.

### 5.3. Structural model

**Fig 2:**
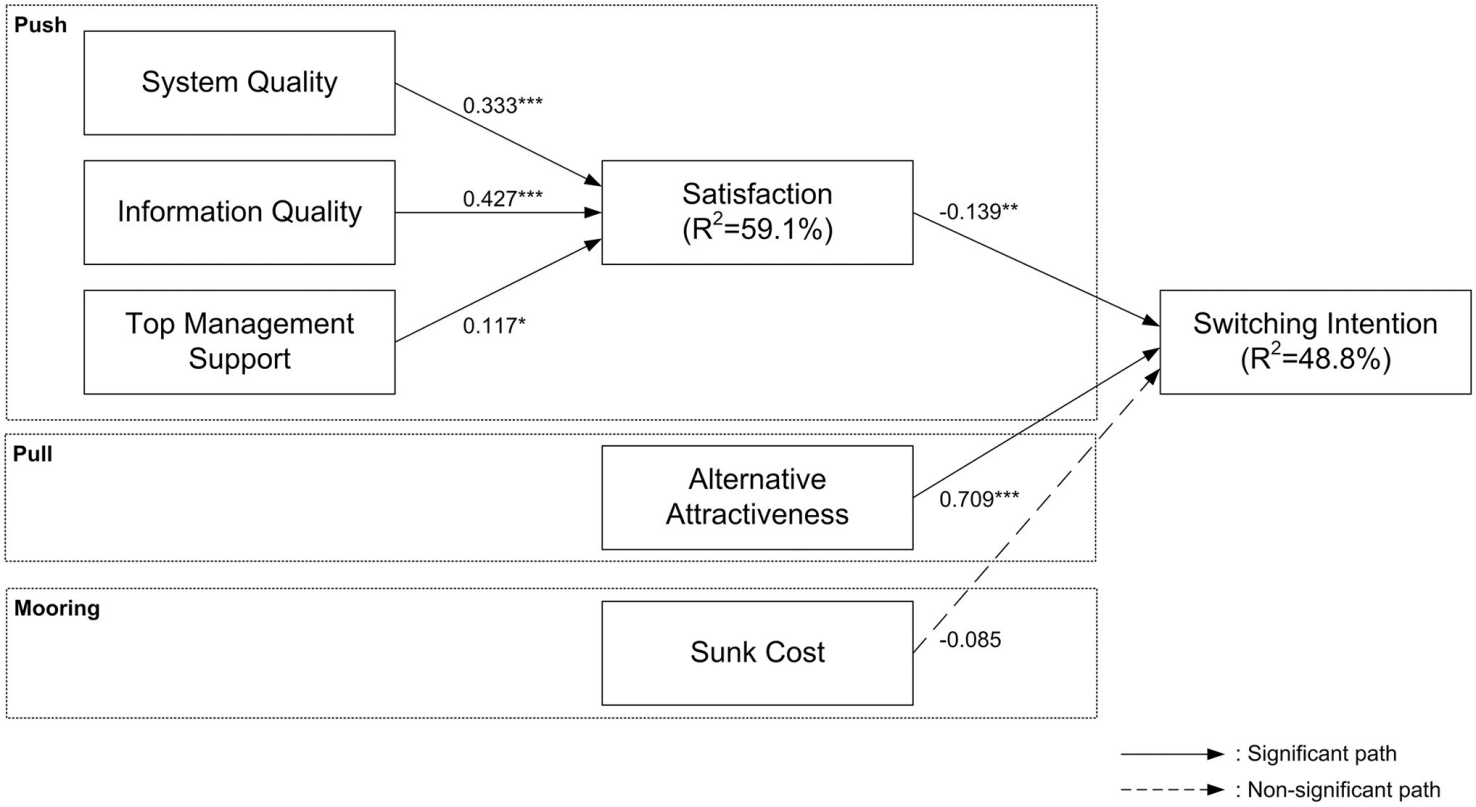
Analysis results. Note: *p<0.05; **p<0.01; ***p<0.001.

The SEM analysis was performed to evaluate the path relationship among the variables. A bootstrap resampling method with 5000 resamples was utilized to determine the significance of the paths within the theoretical framework. The analysis results are shown in Fig 2.

The summary of results is provided in Table 5. Each hypothesis was tested by assessing the cause-effect relationship between each pair of constructs, taking into consideration the calculated coefficient, t-value, and p-value. H1 postulated that system quality positively affects satisfaction. The coefficient value of 0.333 and a t-value of 4.330 (p-value < 0.001) supported this hypothesis. H2 proposed that information quality has a positive effect on satisfaction. With a coefficient value of 0.427 and a t-value of 6.152 (p-value < 0.001), this hypothesis was also supported. H3 argued that top management support positively influences satisfaction. Although the coefficient value was relatively low (0.117), the t-value of 2.184 and a p-value of 0.029 indicated support for this hypothesis. H4 hypothesized that satisfaction negatively influences switching intention. The negative coefficient value of -0.139, along with a t-value of 2.639 (p-value = 0.008), supported this hypothesis, suggesting that higher satisfaction decreases the intention to switch. H5 suggested that alternative attractiveness positively affects switching intention. The strong positive coefficient value of 0.709 and a t-value of 15.795 (p-value < 0.001) strongly supported this hypothesis. However, H6, which posited that sunk costs negatively influence switching intention, was not supported. Despite a negative coefficient of -0.085, the t-value of 1.323 and a p-value of 0.186 indicated that the influence of sunk costs on switching intention was not statistically significant. In summary, five out of six hypotheses were supported by the data.

**Table 5:** Summary of the results.

H	Cause	Effect	Coefficient	t-value	p-value	Hypothesis
H1	System Quality	Satisfaction	0.333	4.870	0.000	Supported
H2	Information Quality	Satisfaction	0.427	6.152	0.000	Supported
H3	Top Management Support	Satisfaction	0.117	2.184	0.029	Supported
H4	Satisfaction	Switching Intention	-0.139	2.639	0.008	Supported
H5	Alternative Attractiveness	Switching Intention	0.709	15.795	0.000	Supported
H6	Sunk Cost	Switching Intention	-0.085	1.323	0.186	Not Supported

Table 6 presents the specific indirect effects through satisfaction. From the analysis, the indirect effects of system quality and information quality on switching intention through satisfaction were significant, indicating that satisfaction is a mediator in these relationships. This suggests that the effect of these constructs on switching intention is partially through the level of satisfaction. The better the quality of the system and information, the higher the satisfaction, which subsequently influences switching intentions. This resonates with the existing literature, highlighting that satisfaction plays a pivotal role in mediating the effects of system and information quality on users’ behaviors [98]. However, the indirect effect of top management support on switching intention via satisfaction was not statistically significant (p > 0.05). This implies that although top management support may affect satisfaction, it does not significantly influence switching intention through the mediation of satisfaction. This suggests that the role of top management support in affecting switching intention may be more complex and may involve other mediational or moderational factors, which warrant further investigation.

**Table 6:** Indirect effects through satisfaction.

Cause	Mediation	Effect	Coefficient	t-value	p-value
System Quality	Satisfaction	Switching Intention	-0.046	2.399	0.016
Information Quality	Satisfaction	Switching Intention	-0.059	2.314	0.021
Top Management Support	Satisfaction	Switching Intention	-0.059	1.599	0.110

## 6. Conclusion

### 6.1. Implications for theory

This study makes several significant theoretical contributions to the literature on ERP user behavior and the PPM model. The first theoretical contribution of our study is the extension of the PPM model into the ERP domain. The PPM model, originating from the field of human geography, has been increasingly applied in various fields such as IS [99–101]. However, its application in understanding the switching intention of ERP users is relatively limited. By applying the PPM model to investigate the factors affecting ERP switching intention, our study contributes to the literature by offering a unique perspective on this critical behavior in the IS field. We provide empirical evidence to demonstrate that the PPM model can be an effective theoretical lens to study ERP switching intention, thus enriching the ERP and IS literature.

The second theoretical contribution of our research is the identification of crucial push, pull, and mooring factors that influence ERP switching intention. Prior research has identified various factors influencing IS switching intention [102–104], but the specific factors that are relevant in the context of ERP systems have been underexplored. Our study has unveiled several key factors such as satisfaction, alternative attractiveness, and sunk cost, which are essential in shaping ERP users’ switching intention. This finding deepens our understanding of the specific factors that drive ERP switching behavior and extends the literature on IS switching intention.

Our third contribution lies in the uncovering of the complex relationships among these push, pull, and mooring factors. This study not only identifies these critical factors but also investigates how they interplay to influence ERP users’ switching intention. For instance, we found that while satisfaction and sunk cost (push and mooring factors, respectively) negatively affect switching intention, alternative attractiveness (a pull factor) has a positive effect. This nuanced understanding of the relationships among the push, pull, and mooring factors provides a more complete picture of the dynamics of ERP switching intention, adding to the richness of the existing literature on IS switching behavior.

The fourth contribution is the confirmation that IS success factors determine the satisfaction of ERP users. The ERP systems can be explicated by the IS success model. The coefficient of information quality on satisfaction was greater than that of system quality on satisfaction, indicating that the quality of information provided by ERP is the most important. Furthermore, it was confirmed that the role of the organization’s management is important in enterprise-wide systems such as ERP. By verifying that IS success factors and management’s support significantly influence satisfaction and switching intention, this study contributes to the literature.

Lastly, this study fills a critical gap in the literature by examining the influence of top management support on ERP user satisfaction and switching intention. Top management plays a critical role in successful ERP implementation and use, as it can shape users’ perceptions and attitudes towards the system [105–107]. Our findings reveal that top management support can enhance user satisfaction with the ERP system and reduce their intention to switch, thereby affirming its importance in the ERP context. Although it is true that top management usually takes strategic decisions such as switching ERP systems, the insights of clerks, assistant managers, and managers can also be invaluable. These employees often directly interact with the system and experience its advantages and shortcomings, thus providing critical feedback influencing top management decisions. However, we concur that our study might have benefitted from including top management team in the sample to get a more holistic view of the switching decision. This study opens up new avenues for future research by demonstrating the relevance and applicability of the expanded PPM model in the ERP context.

### 6.2. Implications for practice

The first practical implication of our study concerns ERP providers. Our findings highlight the critical factors that can drive users to switch from their current ERP system to alternative ones. Specifically, we discovered that satisfaction with the current ERP system is a significant factor that can inhibit users’ switching intentions. This suggests that ERP providers need to ensure their systems are capable of meeting users’ needs and expectations to maintain their satisfaction. For instance, ERP providers could regularly solicit user feedback to identify any issues or shortcomings and promptly address them [108]. By maintaining high user satisfaction, ERP providers can reduce the likelihood of their customers switching to other systems.

Secondly, our study underscores the importance of alternative attractiveness in shaping ERP users’ switching intention. This understanding would be beneficial for service providers, allowing them to compete based on strategies such as the relative strengths of their products and cost advantages. ERP vendors need to understand the solutions potential customers are currently using and identify any problems or limitations. This insight can inform the development of a plan and the building of a sales strategy to encourage customers to switch. This finding has important implications for both incumbent ERP providers and potential new entrants. Incumbent providers need to continually innovate and enhance their systems to meet the evolving demands of users and stay ahead of the competition. Potential new entrants can attract users by offering more attractive alternatives, such as unique features or superior customer service.

Thirdly, the findings showed that information quality and system quality are major determinants of satisfaction. Hence, service providers should manage these qualities at a high level to maintain user satisfaction. Even when a company grows or diversifies its business, the ERP systems must be updated with agility to create an environment that users can easily adapt to and use.

Fourthly, our study highlights the significant role of top management support in shaping ERP users’ satisfaction and, consequently, their switching intention. This finding suggests that top management plays a critical role in the successful use of ERP. To enhance user satisfaction and reduce switching intention, top management could show strong support for the ERP system, such as by providing adequate resources for ERP use and addressing any issues promptly. For example, top management could invest in regular training sessions to enhance users’ skills and confidence in using the ERP system [109–111], which could increase their satisfaction and reduce their intention to switch.

Finally, our study offers valuable insights for policymakers and industry regulators. Our findings on the factors influencing ERP switching intention can inform the development of policies and regulations that promote fair competition in the ERP market. For example, policymakers could introduce regulations that encourage ERP providers to improve their system quality and service, thereby enhancing user satisfaction and reducing switching intention. This would not only benefit users by ensuring they have access to high-quality ERP systems but also stimulate innovation and competition among ERP providers.

### 6.3. Limitations and future research directions

This study presents some limitations and suggestions for future research. Primarily, it assessed ERP switching intention from employees’ viewpoint. However, top management often decides on system changes, suggesting future studies should encompass both these perspectives. Our research also replaced service quality with top management support. Future studies should consider both for a comprehensive analysis. The findings’ generalizability might be limited due to our sample’s specific nature. It is recommended for future research to examine ERP switching intention in different industries and cultural settings. Our focus was primarily on pull, push, and mooring factors within the PPM model, but future research should also explore other factors, such as resistance, technical issues, or vendor-customer dynamics. This paper considered one factor per pulling context and mooring component. Future studies could consider additional factors like peer influence, critical mass, low variety seeking, and prior switching experience. Lastly, the gender imbalance in our sample may have led to potential bias, so future studies should aim for a balanced gender distribution or explore the influence of gender on ERP switching intention explicitly.

## Supporting information

S1 Appendix Appendix A. Lists of Measurement Items.
(DOCX)

S1 Data (ZIP)
