# Supplementary material for: A study of user switching intention for ERP systems based on push-pull-mooring model: Focusing on the important role of information quality for users
Source: PLoS One. 2023 Nov 9;18(11):e0289483. doi: 10.1371/journal.pone.0289483 (PMC10635462; doi:10.1371/journal.pone.0289483)
Supplement: S1 Appendix — Lists of Measurement Items. (DOCX) [file pone.0289483.s001.docx]

# Appendix A. Lists of measurement items

Switching intention is derived from [Kim, Shin [45]](#_ENREF_45).

SWI1: I'm thinking about switching from my existing ERP system to another.

SWI2: There's a good probability I'll switch to a different ERP.

SWI3: I'm resolved to change to a different ERP program.

Satisfaction is derived from [Russell-Bennett, McColl-Kennedy [51]](#_ENREF_51).

SAT1: The existing ERP satisfies all of my needs.

SAT2: ERP has successfully handled any unexpected or urgent scenarios I have encountered.

SAT3: This ERP satisfies my pre-use requirements.

System quality is derived from [Tam and Oliveira [62]](#_ENREF_62).

SYQ1: ERP is fast.

SYQ2: ERP offers appropriate functionality.

SYQ3: ERP is difficult to detect errors or failures.

Information quality is derived from [Urbach and Müller [19]](#_ENREF_19).

INQ1: The information that ERP offers is what I require.

INQ2: The information offered by the ERP is appropriate.

INQ3: The information offered by ERP is accurate.

Top management support is derived from [Hsu, Liu [79]](#_ENREF_79).

TMS1: In recent years, top management has committed to an ERP project's success.

TMS2: Top management publicly stated the goals of the ERP project in recent years.

TMS3: Over the past few years, top management has been involved in every stage of the ERP project.

TMS4: Top management has recently expressed confidence in the cost-effectiveness of ERP.

Alternative attractiveness is derived from [Kim, Shin [45]](#_ENREF_45).

ALA1: I am aware that I have access to more ERP alternatives.

ALA2: Other ERP solutions offer high-quality customer care.

ALA3: Overall, I would be more satisfied with other ERPs

ALA4: There are ERP solutions that I find more appealing than the ones that our company now uses.

Sunk costs are derived from [Jones, Mothersbaugh [55]](#_ENREF_55).

SUC1: In general, I put a lot of effort into my prior use of the current ERP.

SUC2: I put a lot of time and effort into the current ERP.

SUC3: In general, I've invested a lot into using the current ERP.
